# Amino Compounds as Inhibitors of De Novo Synthesis of Chlorobenzenes

**DOI:** 10.1038/srep23197

**Published:** 2016-04-01

**Authors:** Si-Jia Wang, Pin-Jing He, Wen-Tao Lu, Li-Ming Shao, Hua Zhang

**Affiliations:** 1 State Key Laboratory of Pollution Control and Resources Reuse, Tongji University, 1239 Siping Road, Shanghai 200092, P. R. China; 2 Institute of Waste Treatment and Reclamation, Tongji University, 1239 Siping Road, Shanghai 200092, P. R. China; 3 Centre for the Technology Research and Training on Household Waste in Small Towns & Rural Area, Ministry of Housing and Urban-Rural Development of P. R. China, 1239 Siping Road, Shanghai 200092, P. R. China

## Abstract

The inhibitory effects of four amino compounds on the formation of chlorobenzenes (CBzs) - dioxin precursors and indicators, and the inhibitory mechanisms were explored. The results show NH_4_H_2_PO_4_ can decrease the total yields of CBzs (1,2di-CBz, 1,3di-CBz, 1,4di-CBz, penta-CBz and hexa-CBz) by 98.1%±1.6% and 96.1%±0.7% under air and nitrogen flow. The inhibitory effects indicated by the total yields of CBzs follow the order NH_4_H_2_PO_4_ > NH_4_HF_2_ > (NH_4_)_2_SO_4_ > NH_4_Br under air flow and NH_4_H_2_PO_4_ ≈ (NH_4_)_2_SO_4_ ≈ NH_4_HF_2_ >NH_4_Br under nitrogen flow. The inhibition mechanism revealed by thermal analysis that CuCl_2_ was converted to CuPO_3_ by reacting with NH_4_H_2_PO_4_ below 200 °C, which can block the transfer of chlorine and formation of C–Cl bonds at 350 °C. The effects of the other three inhibitors were weaker because their reactions with CuCl_2_, which form other copper compounds, and the reaction of CuCl_2_ with carbon, which forms C–Cl bonds, were almost simultaneous and competitive. Oxygen influenced the yield of CBzs obviously, and the total yield of five CBzs sharply increased with oxygen. Because of their high efficiency, low environmental impact, low cost, and availability, amino compounds - especially NH_4_H_2_PO_4_ - can be utilized as inhibitors of CBzs during incineration.

Incineration is one of the mainstream technologies for treatment of wastes such as municipal solid waste (MSW), medical waste, and other hazardous wastes, due to its volume reduction ability, energy recovery and high efficiency. An important issue for environmental safety and human health is the increased stringency of environmental standards for controlling pollutions. There are still barriers for pollutions control, including toxic chlorinated aromatic compounds and dioxin-like compounds. De novo synthesis^1^,^2^, precursor synthesis^1^,^3^,^4^,^5^ and homogeneous gas synthesis^6^,^7^,^8^,^9^,^10^ have been reported to be the main mechanisms for the formation of polychlorinated dibenzo-*p*-dioxins (PCDDs) and polychlorinated dibenzofurans (PCDFs)^11^. De novo synthesis occurs in the presence of fly ash and chlorine in the post-combustion zone^12^,^13^,^14^, which is believed to contribute more to the generation of PCDD/Fs^11^. Generally, temperatures that favor de novo synthesis range within 300–400 °C^11^ and the role of CuCl_2_ in de novo synthesis is more significant than other metal compounds^9^,^15^,^16^,^17^. Gullett *et al*.^18^ proposed that CuCl_2_ catalyzes Cl_2_ generation by the Deacon reaction between HCl and O_2_, thus promoting dioxin formation. However, the Deacon reaction has been proven not to play such a decisive role^13^,^15^. Addink *et al*.^13^ compared the effects of chlorination with HCl and Cl_2_ on dioxin formation and observed the parallel production of PCDD/Fs. In the presence of oxygen, CuCl_2_ directly provides Cl, a donor for C, thus forming dioxin-like compounds^19^,^20^; this reaction has been confirmed by another study^21^. Takaoka *et al*.^22^ inferred that CuCl_2_ is involved in cyclic conversion via dechlorination and chlorination with oxygen and organic/inorganic chloride. Their theory was reconfirmed by Shao *et al*.^23^ In conclusion, CuCl_2_ is a potential catalyst or Cl donor that promotes the formation of chlorinated aromatic compounds.

On the basis of the known mechanism of dioxin formation, diverse inhibitors have been used in the source control or end-of-pipe removal for dioxin, including nitrogen-containing compounds (NH_3_, urea and (NH_4_)_2_SO_4_)^24^,^25^,^26^,^27^, sulfur-containing compounds (elemental sulfur, SO_2_, (NH_4_)_2_SO_4_ and coal)^25^,^28^,^29^,^30^,^31^, hydroxy-functional groups^23^ and selective catalysts for reduction (SCR)^32^,^33^,^34^,^35^. Among them, the most well-known dioxin inhibitors are highly efficient SCR, such as VO_*x*_/TiO_2_^32^, V_2_O_5_/WO_3_^33^ and TiO_2_/V_2_O_5_/WO_3_^34^,^35^ catalysts, which usually require a complex preparation process and have high cost. Lundin and Jansson^36^ found that the toxic equivalent quantity of PCDF concentration decreased by 75% when the ratio of (NH_4_)_2_SO_4_ to HCl increased from 3:1 to 6:1. The study of Hajizadeh *et al*.^27^ indicated that SO_2_ was more effective than NH_3_ in inhibiting the formation of PCDD/Fs, while the contrast^29^ between (NH_4_)_2_SO_4_ and CO(NH_2_)_2_ was contrary. In recent years, the suppression mechanisms of the formation of chlorinated aromatic pollutants by sulfur-containing and nitrogen-containing compounds have been discussed^20^,^28^,^37^,^38^,^39^, including (1) transformation of Cl_2_ to HCl, (2) sulfonation of dioxins or precursors and (3) conversion of metal chlorides (CuCl_2_ or FeCl_3_) with high catalytic activity to inert compounds. Yan *et al*.^29^ attributed the reduction of PCDD/Fs production by urea to the reaction of ammonia with active oxidant Cl_2_. Shao *et al*.^40^ and Fujimori *et al*.^28^ proposed that SO_2_ and H_2_O can convert CuCl or CuCl_2_ to CuSO_4_ or CuO by detecting the residues, which was also proved by the thermodynamic equilibrium calculation^41^. However, the fundamental information that enables an understanding of these mechanisms is still insufficient.

Amino compounds decompose at low temperature, and their reactions with CuCl_2_ at low temperature are thermodynamically favorable, thereby they are generally advantageous in inhibiting CuCl_2_ from donating Cl to form C–Cl bonds. In this study, we studied the inhibitory effects and mechanisms of four amino compounds on chlorobenzenes (CBzs), which are important precursors^42^ and indicators of dioxin^43^. Except (NH_4_)_2_SO_4_, the other three were used as inhibitor for the first time and their effects were compared with (NH_4_)_2_SO_4_. Thermal analysis, which is beneficial to distinguishing the characteristic thermal reactions between inhibitors and model fly ash, was conducted combining with the simulated experiments, to reveal which one was more effective in inhibiting CBzs formation and why (mechanism).

## Results

### Effect of inhibitors on CBzs formation

**Figure 1:**
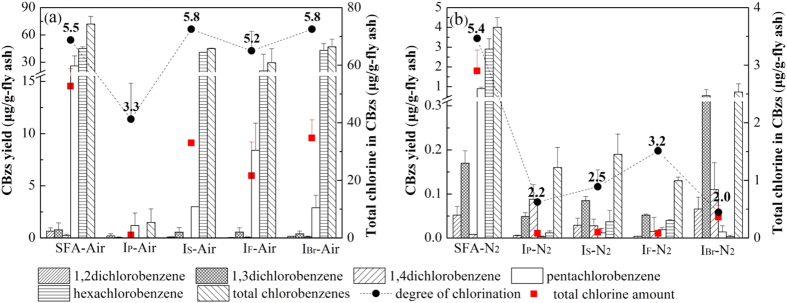
CBzs yields, total chlorine in CBzs and the degree of chlorination under air (a) and nitrogen (b) flow.

**Figure 2:**
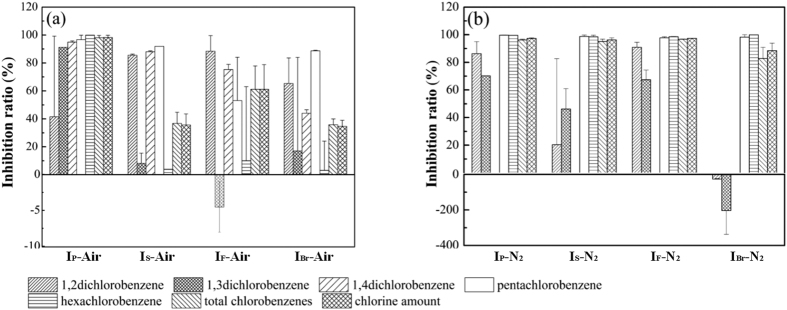
Inhibition ratios of CBzs and total chlorine in CBzs under air (a) and nitrogen (b) flow (The concentration of 1,4-CBz from SFA-N_2_ was below the detection limit, thus the inhibition ratios were not calculated. The slight increases of 1,4-CBz in I_P_-N_2_, I_s_-N_2_, I_F_-N_2_, I_Br_-N_2_ were found, i.e., 0.08, 0.02, 0.007, and 0.1 μg/g-fly ash compared with SFA-N_2_).

The yields of the five CBzs and their total value on the mass basis of the SFA (μg/g-fly ash) are shown in Fig. 1. The total chlorine in the CBzs (equation (1)) and the degree of chlorination (equation (2)) were calculated from these yields (Fig. 1). The inhibition ratios for CBzs formation with each inhibitor were calculated through equation (3) and presented in Fig. 2.


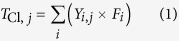



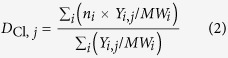



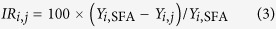


where *i* is the type of CBzs, *j* is the type of sample, and *T*_Cl, *j*_ is the total amount of chlorine in the CBzs on mass basis of the SFA sample *j* (μg/g-fly ash), *Y*_*i, j*_ and *Y*_*i*, SFA_ are the yields of CBz *i* on the mass basis of the SFA sample *j* and SFA, respectively (μg/g-fly ash), *F i* is the mass fraction of chlorine in CBz *i* (dimensionless), *D*_Cl, *j*_ is the degree of chlorination of sample *j* (dimensionless), *n*_*i*_ is the number of chlorine atoms in the CBz molecule *i* (dimensionless), *MW*_*i*_ is the molecular weight of *i* (mol/g), and *IR*_*i, j*_ is the inhibition ratio of CBz *i* for the sample *j* (%).

Under air flow, the yields of low-chlorinated CBzs (1,2di-CBz, 1,3di-CBz, and 1,4di-CBz) were much lower than those of high-chlorinated CBzs (penta-CBz and hexa-CBz) in SFA, similar to the trends for the NH_4_H_2_PO_4_ (I_P_), (NH_4_)_2_SO_4_ (I_S_), NH_4_HF_2_ (I_F_), and NH_4_Br (I_Br_) runs. This result indicated that high-chlorinated CBzs formed more easily than low-chlorinated CBzs did. A study by Fujimori *et al*.^28^ also showed similar distribution ratios, but the absolute yield of each CBz in the present study was higher because of the larger amount of CuCl_2_·2H_2_O added, which is necessary for determining the mechanism. As shown in Fig. 1, chlorine from the two CBzs accounted for a large proportion of the total chlorine because of the high yield and high fraction of chlorine in penta-CBz and hexa-CBz.

Compared with that of SFA, the total yield of CBzs and the total chlorine in CBzs decreased by various degrees upon addition of inhibitors under air flow, especially in the case with NH_4_H_2_PO_4_ addition. The yields of low-chlorinated CBzs (1,2di-CBz, 1,3-CBz, 1,4-CBz) and high-chlorinated CBzs (penta-CBz, hexa-CBz) for the SFA–Air mixture were 1.7 and 71 μg/g-fly ash respectively, which significantly decreased to 0.28 and 1.2 μg/g-fly ash for the I_P_–Air mixture. Formation of all CBzs was inhibited by 41%–100%. The total chlorine from CBzs also decreased, and the degree of chlorination changed from 5.5 to 3.3. The other three inhibitors had different effects on the yields of different CBzs. To evaluate the reduction of CBzs formation, NH_4_H_2_PO_4_ was compared with those inhibitors. Kuzuhara^25^ found that the amount of formed PCDD/Fs decreased significantly upon addition of ammonia or urea. A possible mechanism for the suppression is a competing reaction of organic compounds with NH_*i*_ and CN radicals, which are produced from urea or ammonia decomposition. However, they suggested that further studies are necessary to evaluate the effect of these compounds on the behavior of copper and the role in the de novo synthesis.

With I_S_ under air flow, the total yield of CBzs decreased by 37%, and the penta-CBz yield decreased by 92% to 3.0 μg/g-fly ash. The degree of chlorination did not decrease, but the total chlorine from CBzs declined by 36%. In Yan’s^29^ study, (NH_4_)_2_SO_4_ reduced the yield of PCDD/Fs in the gas phase by about 93% (about 60% PCDDs and 98% PCDFs); when gaseous SO_2_ was used^28^, the yields of CBzs, PCDDs, and PCDFs were reduced by about 50%, 30%, and 50%, respectively. Thus, (NH_4_)_2_SO_4_ and SO_2_ are effective inhibitors of dioxin and CBzs formation. The mechanism of (NH_4_)_2_SO_4_ inhibition of CBzs de novo synthesis is discussed later. Some S-containing or N-containing compounds showed inhibitory effects on dioxin synthesis, such as ethylenediaminetetraacetic acid, nitrilotriacetic acid, and Na_2_S^44^. These effects were explained owing to the interaction between inhibitors and catalysts such as Cu.

Based on the inhibitory effects of the amino compounds on CBzs formation, NH_4_HF_2_ and NH_4_Br were selected to study their potential effects on controlling CBzs. The results show that the inhibition effect of NH_4_HF_2_ is similar to that of (NH_4_)_2_SO_4_ on the synthesis of low-chlorinated CBzs and penta-CBz, while NH_4_HF_2_ is better on the hexa-CBz inhibition. The degree of chlorination in I_F_ decreased from 5.5 to 5.2 with inhibitions on penta-CBz and hexa-CBz. The effect of NH_4_Br was smaller than that of the other three, but it still reduced the formation of all CBzs and the total yield of CBzs by 3%–88.7%.

Under nitrogen flow, CBzs yields in all cases were very low compared with those obtained under air flow. This difference is due to the difficulty of C–C bond scission in the absence of oxygen, which leads to formation of aromatic compounds^22^, in agreement with the study of Yan^29^. Upon addition of any of the inhibitors, the total yield of CBzs decreased along with the significant decrease in the yield of penta-CBz and hexa-CBz (Fig. 2b). The concentration of 1,4-CBz from SFA-N_2_ was below the detection limit, thus the inhibition ratios were not calculated. The slight increases of 1,4-CBz in I_P_-N_2_, I_s_-N_2_, I_F_-N_2_, I_Br_-N_2_ were found, i.e., 0.08, 0.02, 0.007, and 0.1 μg/g-fly ash compared with SFA-N_2_.

Chlorobenzenes are closely associated with PCDD/Fs production^43^,^45^,^46^. The amounts of the most toxic congeners, 2,3,7,8-TCDD and 2,3,4,7,8-PeCDF, with respect to amounts of penta-CBz have high correlation coefficients^45^. In this study, all additives showed clear inhibitory effects on CBzs synthesis; they decreased the total CBzs yields under air or nitrogen flow (Fig. 2c,d). The inhibitory effects on CBzs production follow the order NH_4_H_2_PO_4_ > NH_4_HF_2_ > (NH_4_)_2_SO_4_ > NH_4_Br under air flow and NH_4_H_2_PO_4_ ≈ (NH_4_)_2_SO_4_ ≈ NH_4_HF_2_ >NH_4_Br under nitrogen flow. Many studies reported the effects of N-containing and S-containing compounds on PCDD/Fs formation, but less on CBzs formation. NH_4_H_2_PO_4_ in this study showed significantly higher inhibition on CBzs formation than gaseous SO_2_ by Fujimori *et al*.^28^, while (NH_4_)_2_SO_4_ showed a little less inhibition effect. In Hajizadeh’s study, both SO_2_ and NH_3_ were effective in inhibiting the formation of PCDD/Fs^27^, and the effect of SO_2_ was more significant than that of NH_3_. Even though some controversies existed in the synergistic or competitive effect on PCDD/Fs inhibition by S-containing and N-containing compounds, (NH_4_)_2_SO_4_ as a complex of S and N has been confirmed to have the restraint effect on PCDD/Fs^29^ as well as CBzs in this study. In the contrast of (NH_4_)_2_SO_4_ with NH_4_H_2_PO_4_ and NH_4_HF_2_, especialy NH_4_H_2_PO_4_, showed the significant increase of inhibition efficiency on CBzs. Thus, the amino compounds can decompose and produce reactive radicals that are highly-efficient on suppressing CBzs.

### Thermal analysis of the SFA samples

As discussed above, the four amino compounds influence the profile of CBzs. However, the reason why they showed different inhibitory results, and how they affected CBzs formation were not clear, which might be related to their thermochemistry. To better understand the inhibition mechanism and to observe the physical transformation or chemical reactions, thermal analysis using TGA and DSC was conducted. The fraction of CuCl_2_ was increased to higher than that in real fly ash, so that the important physical-chemical changes could be observed together with the change of CBzs formation. Commonly used (NH_4_)_2_SO_4_ was included in this studies for the comparison.

**Figure 3:**
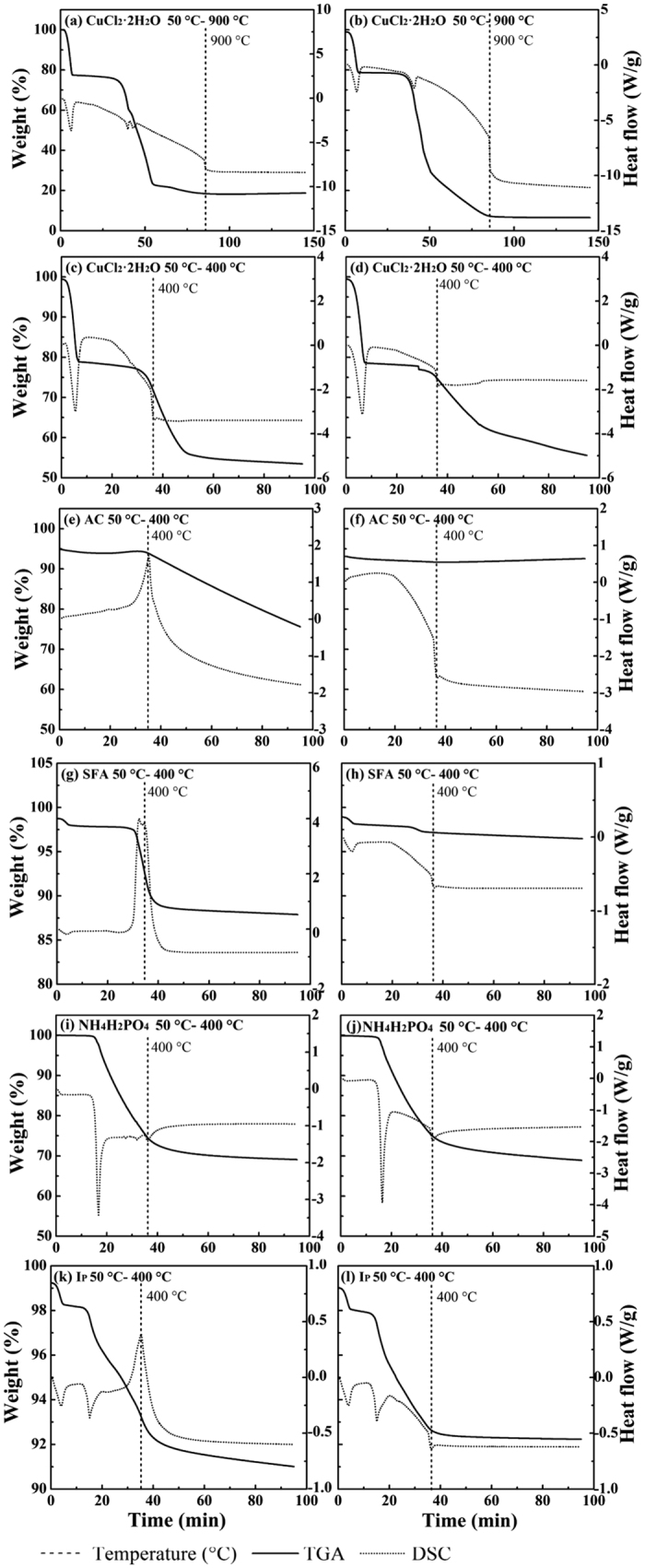
TGA-DSC of CuCl_2_·2H_2_O, AC, SFA, NH_4_H_2_PO_4_ and I_P_ under the air (a,c,e,g,i,k) and nitrogen (b,d,f,h,j,l) flow.

**Figure 4:**
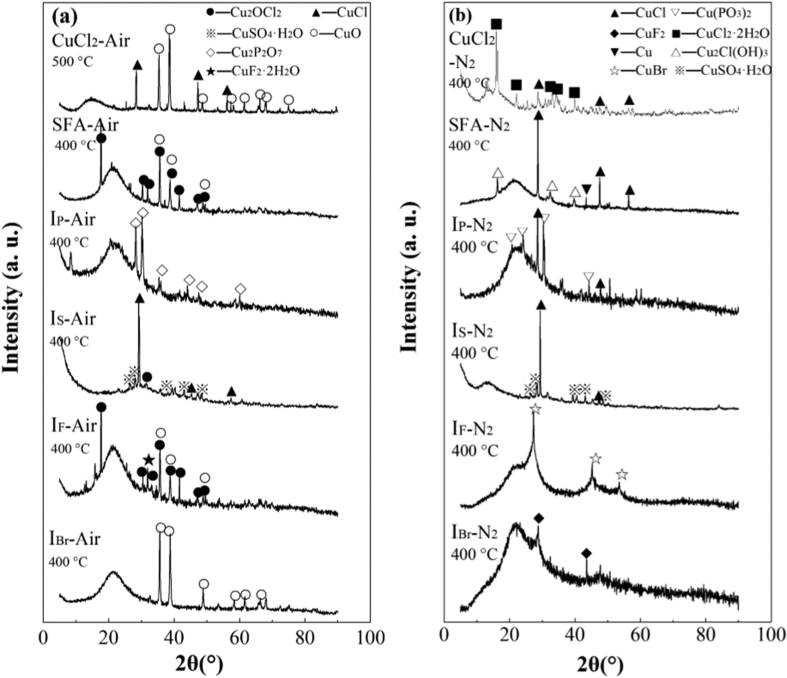
XRD patterns of the residues under air (a) and nitrogen (b) flow.

As shown in Fig. 3a,b, CuCl_2_·2H_2_O gradually underwent dehydration (21 wt.%) at 50–100 °C and started to lose weight at 343 °C (air) or 348 °C (nitrogen). When the temperature reached 900 °C, the weight decreased by about 55% under air flow and by 65% under nitrogen flow. Under both atmospheres, one endothermic peak was produced at 441–445 °C. Another appeared at 471 °C only in the presence of oxygen. Under nitrogen flow at 350 °C, CuCl_2_ dechlorinated, forming CuCl, which volatilized at 438–441 °C. Therefore, the residue formed at 400 °C and detected by XRD includes CuCl only (Fig. 4b). A similar finding was reported by Liu *et al*.^47^ Under air flow, the residue obtained at 400 °C includes CuCl only (data not shown), whereas that obtained at 500 °C consisted of CuCl and CuO (Fig. 4a), which is in accord with the dechlorination observed by Takaoka *et al*.^22^ The endothermic peak on the DSC curve and the smaller weight loss (10% less) compared with that under nitrogen flow suggest that oxygen oxidizes CuCl to CuO. The reactions and corresponding temperatures are indicated in equations (4)–(7), 5, 6, 7. In the absence of other chlorine source, CuCl_2_ produces chlorinated aromatic compounds, behaving as a Cl resource, transferring Cl to the C surface, and bonding with C. Takaoka *et al*.^16^ and Fujimori *et al*.^28^ found that the thermochemical conversion of CuCl_2_ and thermal reaction of CuCl_2_ with SO_2_ occurred at below 300 °C and 280–350 °C respectively. In this study, the thermal conversion observed by DSC gave a direct and specific temperature that CuCl_2_ decomposed, which is propitious to mechanism study.


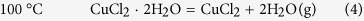



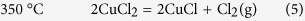



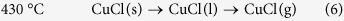



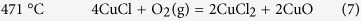


To identify the characteristic DSC peaks, AC and silica were respectively analyzed by TGA and DSC, respectively. In the presence of oxygen, AC started to combust at 400 °C (Fig. 3e), producing an intense exothermic peak. This also quickly increased the interior temperature, while the weight and heat flow remained stable under nitrogen flow (Fig. 3f). As no significant changes in TGA and DSC curves for silica were observed, they are not displayed.

The calculated weight loss of SFA consisting of AC and CuCl_2_·2H_2_O under air flow (according to the weight loss in TGA and mass fraction) was 9.5%, and the actual weight loss was 15%. This difference is probably caused by the greater volatilization of CuCl at higher internal temperature and the greater emission of aromatic compounds in the presence of oxygen. Under nitrogen flow, there was almost no difference in weight losses. XRD patterns (Fig. 4) show that the residue under air flow includes Cu_2_OCl_2_ and CuO and that under nitrogen flow includes CuCl, Cu, and Cu_2_Cl(OH)_3_. The presence of CuO under air flow contradicts with the results of thermal analysis in the 50–400 °C range (Fig. 3c), probably because the combustion of carbon in this system can increase the interior temperature.

## Discussion

According to Fig. 3i,j, NH_4_H_2_PO_4_ started to decompose at 203 °C under either air or nitrogen flow. An endothermic peak appeared at almost the same temperatures. NH_4_H_2_PO_4_ decomposed into gaseous NH_3_ and H_2_O and solid-phase HPO_3_, as shown by the weight loss (~30%) and by thermodynamic calculation (equation (8)). The weight loss for I_P_ under air flow was calculated by summing the respective weight losses of silica, AC, CuCl_2_·2H_2_O, and NH_4_H_2_PO_4_. The sum should be ~20% if there was no interaction between CuCl_2_ and NH_4_H_2_PO_4_. The real weight loss was 10% less than the calculated value, indicating the occurrence of interaction. In addition, endothermic peaks were produced at 100 °C and 191 °C under both atmospheres (Fig. 3k,l) and an exothermic peak was produced at 400 °C under air flow. Both indicate dehydration, a reaction between NH_4_H_2_PO_4_ and CuCl_2_, and carbon combustion. According to the XRD results (Fig. 4), the products included Cu_2_P_2_O_7_ (Cu(PO_3_)_2_·CuO) (air), as well as CuCl and Cu(PO_3_)_2_ (nitrogen), which suggest that CuCl_2_ reacted with NH_4_H_2_PO_4_ (equation (9)). In contrast to SFA, CuCl_2_ converted to Cu(PO_3_)_2_ at a lower temperature instead of transferring Cl to C; thus, CBzs formation was restrained significantly. This not only explains the mechanism, but also suggests that CuCl_2_ was the main Cl source. The diminution of the total chlorine in CBzs when NH_4_H_2_PO_4_ was applied was caused by the decrease in the yield of CBzs and the degree of chlorination.


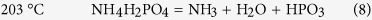



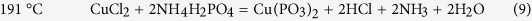


**Figure 5:**
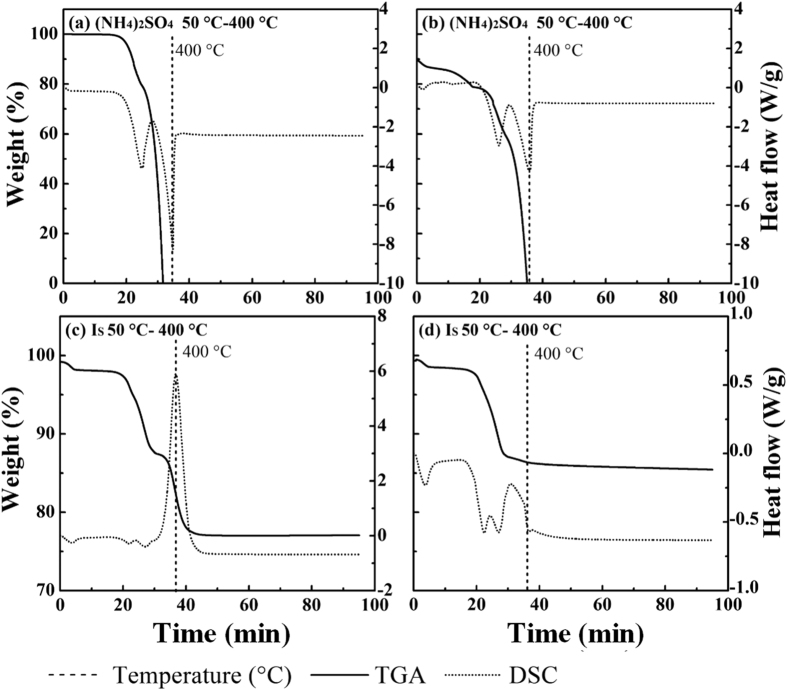
TGA-DSC of (NH_4_)_2_SO_4_ and I_S_ under air (a,c) and nitrogen (b,d) flow.

The suppression effects of (NH_4_)_2_SO_4_, (NH_4_)_2_S_2_O_3_, CO(NH_2_)_2_S, and SO_2_ on the formation of PCDD/Fs have been studied^23^,^25^,^28^,^29^,^36^, but the mechanisms with these inhibitors are not as clear as that with SO_2_. One proposed mechanism is the conversion of copper into non-reactive sulfates^30^. Partial sulfation of CuCl_2_ by SO_2_ in the presence of O_2_ to CuSO_4_ and Cl_2_ has been reported^28^. Below 400 °C in this study (Fig. 5), (NH_4_)_2_SO_4_ completely decomposed into gaseous NH_3_, H_2_O, and SO_3_, producing endothermic peaks at 293 °C (air) and 297 °C (nitrogen) (equation (10)). Upon addition of (NH_4_)_2_SO_4_ to SFA, endothermic peaks were produced at 100, 260, and 312 °C under air flow, as well as at 100, 263, and 312 °C under nitrogen flow. An exothermic peak at 400 °C was also produced under air flow. The characteristic peaks at 260–263 °C under both atmospheres do not correspond to the constituents of I_S_, indicating that a reaction between CuCl_2_ and remaining (NH_4_)_2_SO_4_ occurred with this endothermic phenomenon (equation (11)). When there was no interaction between SFA and (NH_4_)_2_SO_4_, the calculated weight loss was 9% less than the experimental value, suggesting that some products remained in the residues. The XRD results show Cu_2_OCl_2_, CuO, and weak CuSO_4_·H_2_O peaks, which indicate that dechlorination and sulfation were simultaneous, with dechlorination being dominant. Therefore, the inhibitory effect was weaker than that of NH_4_H_2_PO_4_. Although the total yield of CBzs and the total chlorine in CBzs decreased, the degree of chlorination did not decline because hexa-CBz easily formed with the stable structure.


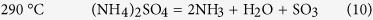



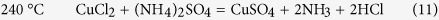


**Figure 6:**
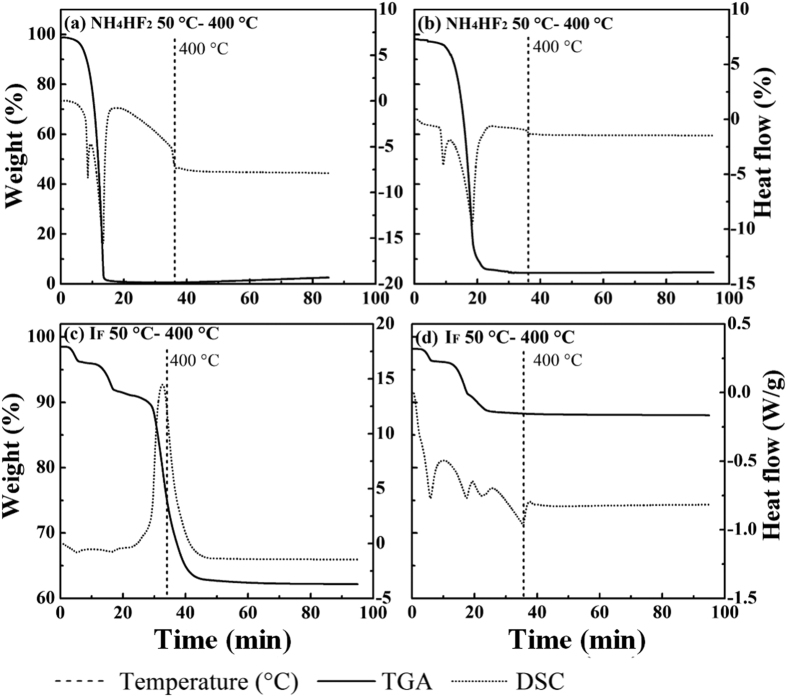
TGA-DSC of NH_4_HF_2_ and I_F_ under air (a,c) and nitrogen (b,d) flow.

NH_4_HF_2_ easily decomposed as shown in Fig. 6. It transformed into gas from 80 to 270 °C under both air and nitrogen flow (equation (12)). There was a characteristic endothermic peak at 130 °C signifying this decomposition process. When NH_4_HF_2_ was added, faint endothermic peaks were produced at 95, 205, and 400 °C under air flow, as well as at 98 and 213 °C under nitrogen flow. Multiple-step weight losses occurred at temperature ranges of 72–114 °C, 158–216 °C, and 325–400 °C under air flow, as well as at 75–112 °C and 159–297 °C under nitrogen flow. These weight losses correspond to hydration, a reaction between CuCl_2_ and NH_4_HF_2_, and carbon combustion. The residue (Fig. 4) formed under air flow was a mixture of CuO, Cu_2_OCl_2_, and CuF_2_, whereas that formed under nitrogen flow consisted of CuF_2_. The reaction temperature for equation (13) was higher than that for NH_4_HF_2_ decomposition. In addition, the amount of the functional radical of F^−^ did not exceed that of Cu^2+^, thus decreasing the degree of conversion and the inhibitory effect. The yields of CBzs and the degree of chlorination of I_F_ were lower than those of I_P_, indicating low degree of conversion.


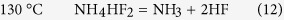



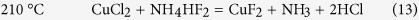


**Figure 7:**
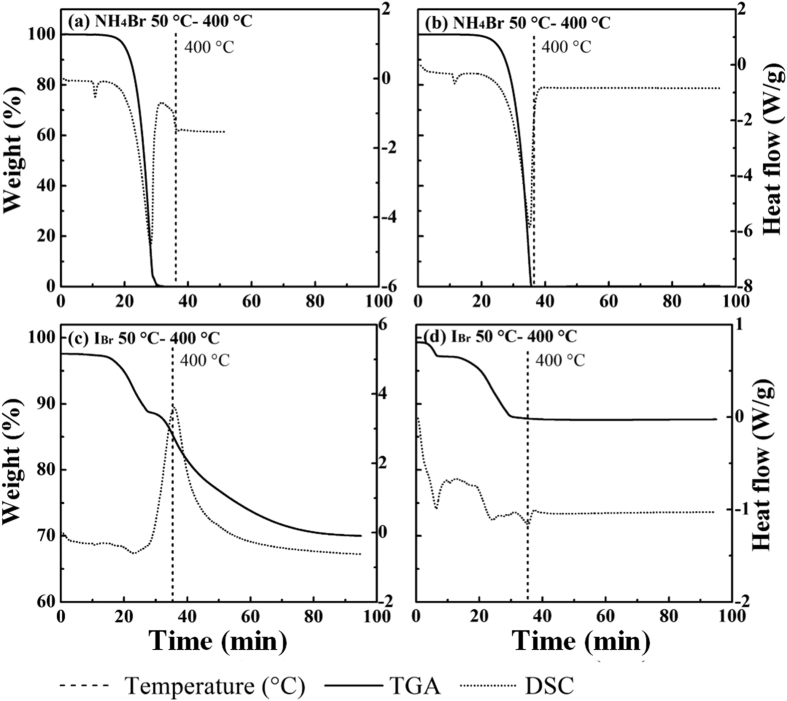
TGA-DSC of NH_4_Br and I_Br_ under air (a,c) and nitrogen (b,d) flow.

Similar to NH_4_HF_2_, NH_4_Br is an unstable amino compound below 400 °C, producing an endothermic peak at 155 °C (Fig. 7). From 180 to 375 °C, NH_4_Br started to decompose (equation (14)) and lost weight completely. As the TGA curves show, two-step weight losses occurred at 179–313 °C and 350–400 °C under air flow, and one-step loss occurred at 176–350 °C under nitrogen flow when NH_4_Br was added to SFA. The DSC curves obtained under air flow show weak endothermic peaks at 144 and 268 °C and intense peaks at 400 °C. Those obtained under nitrogen flow have peaks at 100, 146, and 277 °C. New peaks at 268 °C (air) and 277 °C (nitrogen) are characteristic of the product I_Br_, indicating that there was an interaction between SFA and NH_4_Br. This interaction is also evidenced by comparison between the actual and calculated weight losses. The residues obtained under air flow (Fig. 3) and under nitrogen flow consisted of CuO and CuBr, respectively. Consistent with the results of thermal analysis and XRD, CuCl_2_ could react with NH_4_Br during its decomposition (equation (15)). As these two changes occurred under almost same temperature ranges, the inhibitory effect decreased.


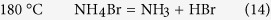



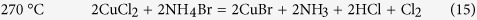


## Methods

### Materials

The mass composition of the simulated fly ash (SFA) in each experiment is shown in Table 1. The inhibitors were added at Cu/H_2_PO_4_^−^, Cu/SO_4_^2−^, Cu/HF_2_^−^ and Cu/Br^−^ molar ratios of 0.5 and then blended with other constituents.

Before use, activated carbon (AC) was milled to <150 μm and heated at 600 °C under nitrogen flow to allow desorption. Amorphous silica milled to <150 μm was used as a matrix to avoid high intensities of diffraction peaks. Analytical-reagent-grade AC, CuCl_2_·2H_2_O and the amino compound inhibitors (NH_4_H_2_PO_4_, (NH_4_)_2_SO_4_, NH_4_HF_2_, NH_4_Br), as well as reagent-grade 1,3,5-tribromobenzene (internal standard), were purchased from Sinopharm Chemical Reagent Co. Ltd. (China). Guaranteed reagent grade CBzs mixture standard (1,2di-CBz, 1,3di-CBz, 1,4di-CBz, penta-CBz, and hexa-CBz), HPLC-grade hexane, acetone, and XAD-II resin (Supelco) were purchased from Sigma-Aldrich Company. Florisil solid-phase extraction columns were obtained from ANPEL Laboratory Technologies (Shanghai) Incorporated.

### Simulation experiments

The experiments were carried out in duplicate with a tube furnace, equipped with an XAD-II resin tube and two impingers in series (the first being empty and the second filled with 100 mL of hexane), which absorbed CBzs in the flue gas. The temperature was maintained at 400 °C, and the gas flow (air and nitrogen) was set at 1000 mL/min. Each quartz crucible was filled with 5 g of the sample, placed in the middle chamber in 30 s, and then held there for 60 min. Then the residues in the crucibles were cooled down under the air or nitrogen flow, and were collected for X-ray diffraction measurement (XRD, D8 Advance, Bruker, Germany) using Cu Kα radiation.

The XAD-II resin was collected and extracted with 100 mL of hexane at 140 °C for 5 h by using an automatic Soxhlet extractor (Soxtec TM 2050, Foss, USA). The extract was then mixed with the absorption solvent and concentrated to about 10 mL by rotary vacuum evaporation (R1002B, Senco, China) in water bath at 85 °C. Then nitrogen evaporation (N-EVAD, Organmation, USA) with 2 mL/min N_2_ flow was used to further concentrate the liquid to 2 mL. The concentrate was loaded into a Florisil solid-phase extraction cartridge (Visiprep DL SPE, supelco, USA) for purification, and then eluted by 10 mL of the mixture of acetone and hexane (1/9, v/v). The elute was concentrated by the nitrogen evaporation to 1 mL before analysis by gas chromatography (GC) (Trace, Thermo, USA).

The GC was equipped with a HP-5MS column (30 m × 0.25 mm ID) (Agilent, USA) and an electron capture detector. The oven temperature program was set at: 40 °C for 1.5 min, 10 °C/min to 100 °C with 3 min holding time, 10 °C/min to 240 °C with a final hold of 1 min. The carrier gas was helium (30 mL/min), the detector temperature and transfer line temperature were set at 300 °C and 250 °C respectively. The analysis of 1,2di-CBz, 1,3di-CBz as well as 1,4di-CBz were splitless, penta-CBz and hexa-CBz were split with split ratio 10:1. The yields of 1,2di-CBz, 1,3di-CBz, 1,4di-CBz, penta-CBz, and hexa-CBz were calculated on the mass basis of fly ash. The recovery ratios for the analysis of CBzs in the resins ranged within 70%–130%.

Thermogravimetric analysis (TGA) and differential scanning calorimeters (DSC) (Q600 SDT, TA instrument, USA) were used for thermal analysis of the samples. Heating was done at a rate of 10 °C/min from 50 °C to specified values, at which the temperature was maintained for more than 60 min. The flow rate of air or nitrogen was adjusted to 100 mL/min.

## Additional Information

**How to cite this article**: Wang, S.-J. *et al*. Amino Compounds as Inhibitors of De Novo Synthesis of Chlorobenzenes. *Sci. Rep.* **6**, 23197; doi: 10.1038/srep23197 (2016).

## Figures and Tables

**Table 1:** Mass fraction of the compounds used in the experimental runs.

Sample	Parameters	CuCl_2_·2H_2_O (wt.%)	Activated carbon (wt.%)	Silica (wt.%)	Inhibitors (wt.%)			
					NH_4_H_2_PO_4_	(NH_4_)_2_SO_4_	NH_4_HF_2_	NH_4_Br
SFA-Air	Air, 400 °C	10.0	20.0	70.0	–	–	–	–
I_P_-Air	Air, 400 °C	10.0	20.0	56.5	13.5	–	–	–
I_S_-Air	Air, 400 °C	10.0	20.0	54.3	–	15.7	–	–
I_F_-Air	Air, 400 °C	10.0	20.0	66.6	–	–	3.4	
I_Br_-Air	Air, 400 °C	10.0	20.0	58.5	–	–	–	11.5
SFA-N_2_	N_2_, 400 °C	10.0	20.0	70.0	–	–	–	–
I_P_-N_2_	N_2_, 400 °C	10.0	20.0	56.5	13.5	–	–	–
I_S_-N_2_	N_2_, 400 °C	10.0	20.0	54.3	–	15.7	–	–
I_F_-N_2_	N_2_, 400 °C	10.0	20.0	66.6	–	–	3.4	
I_Br_-N_2_	N_2_, 400 °C	10.0	20.0	58.5	–	–	–	11.5
